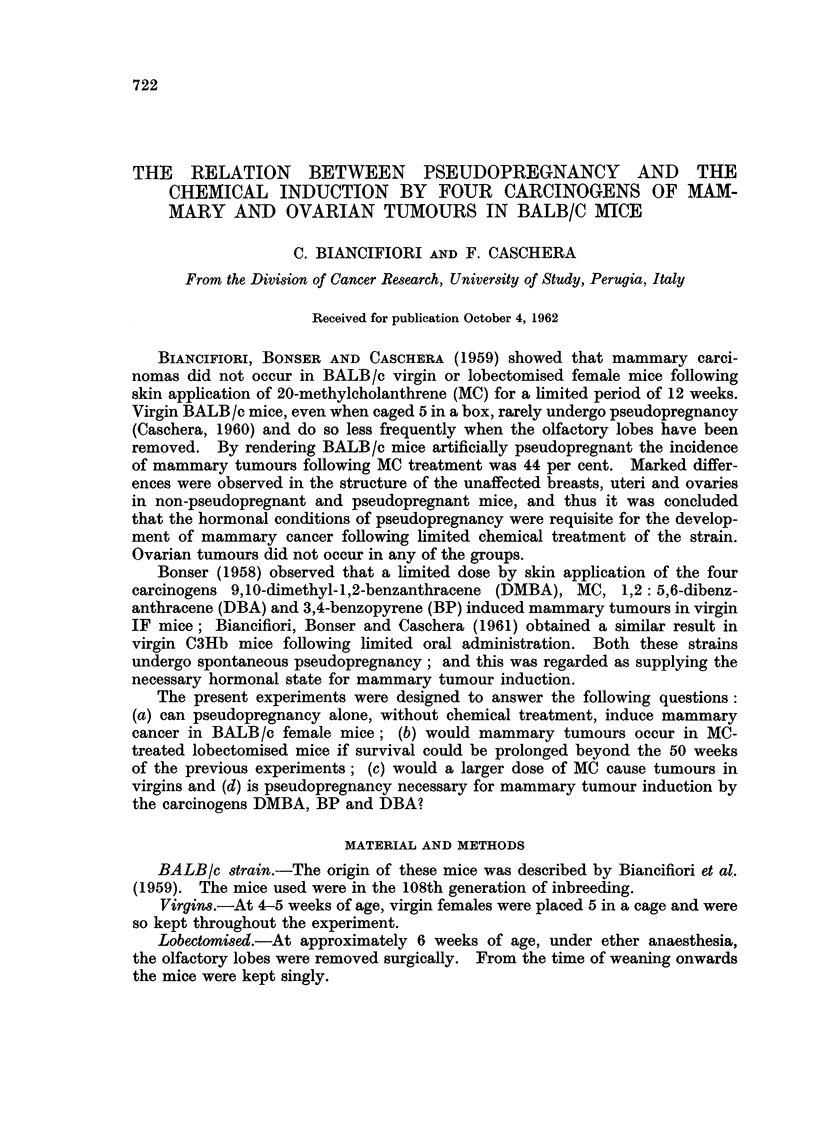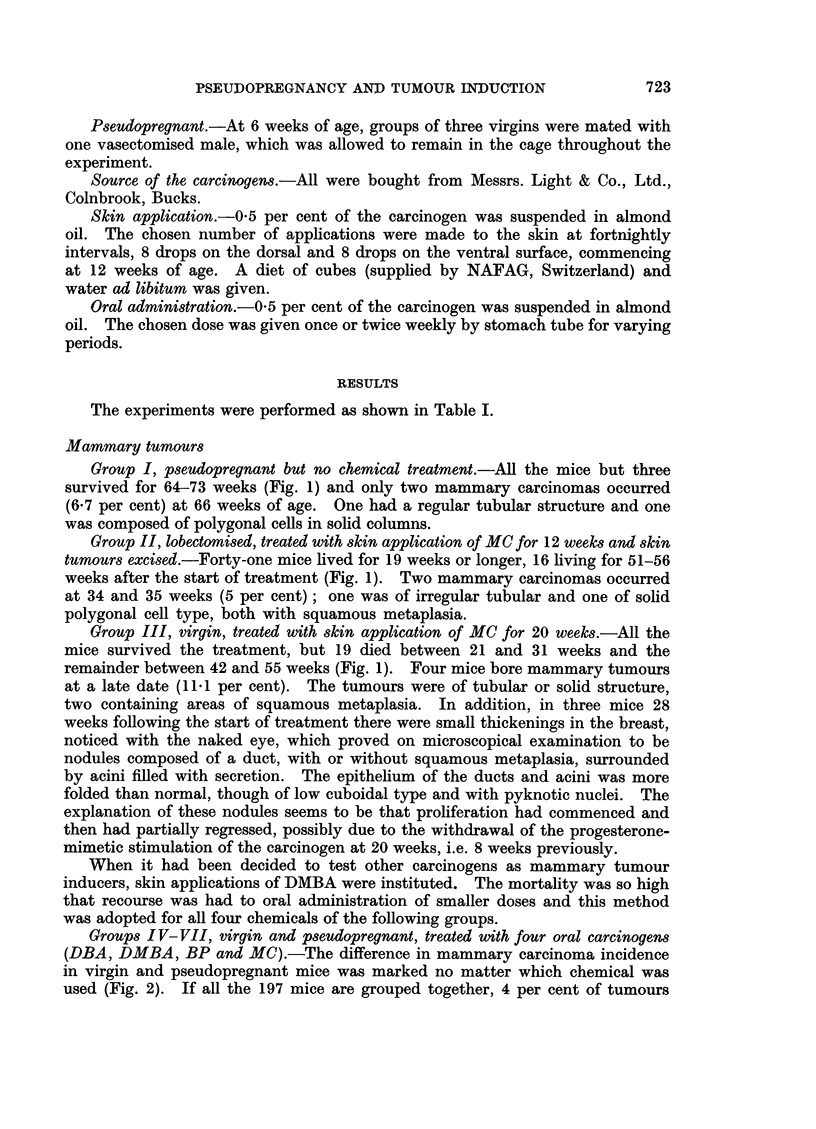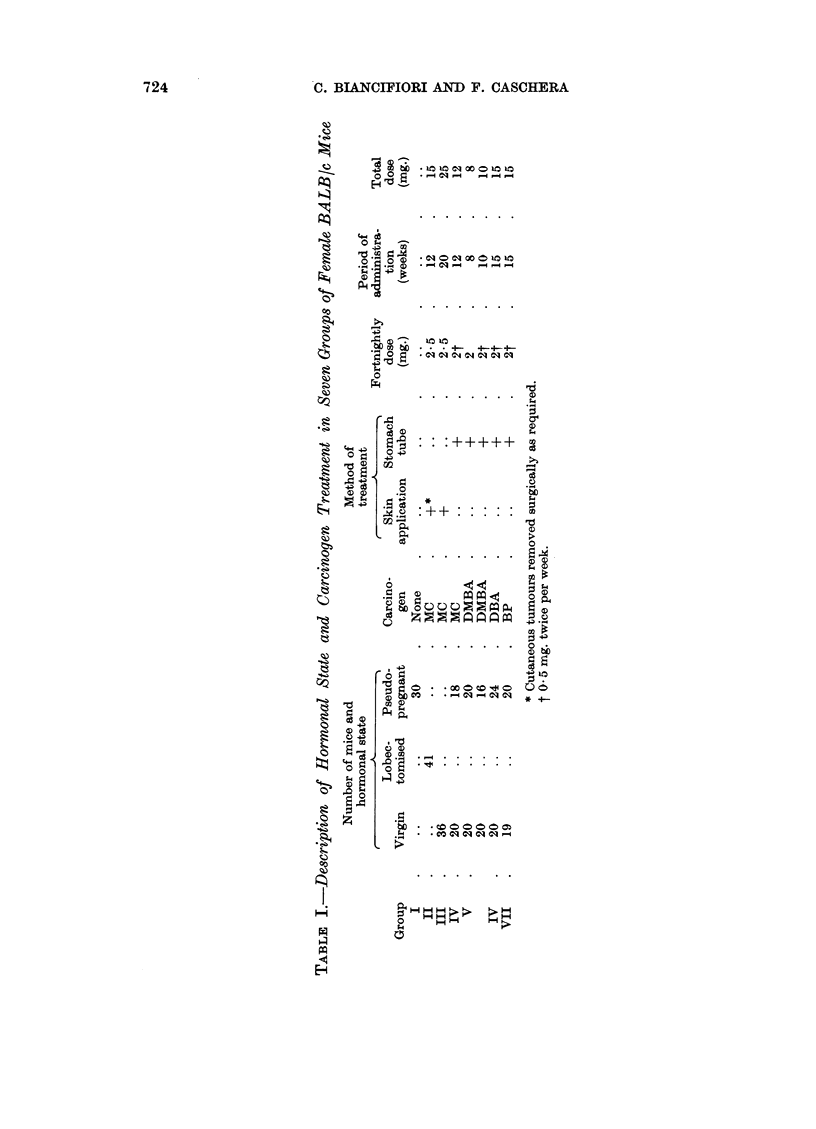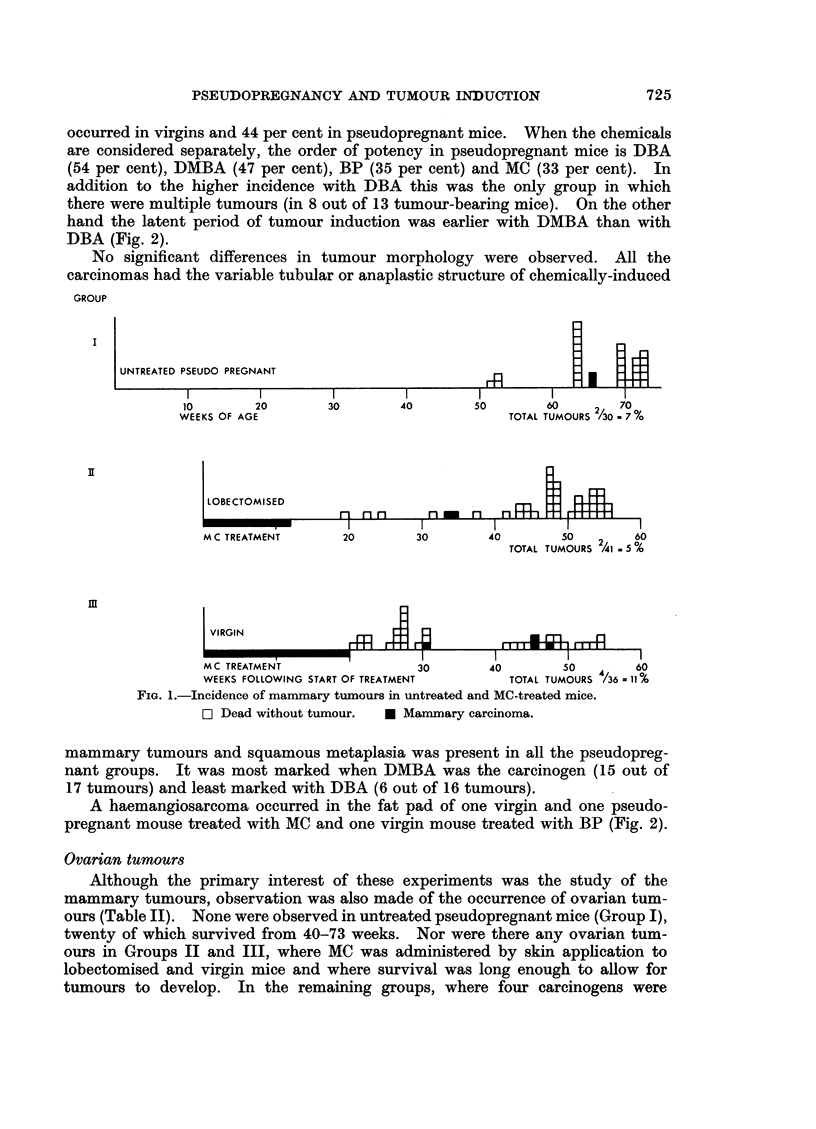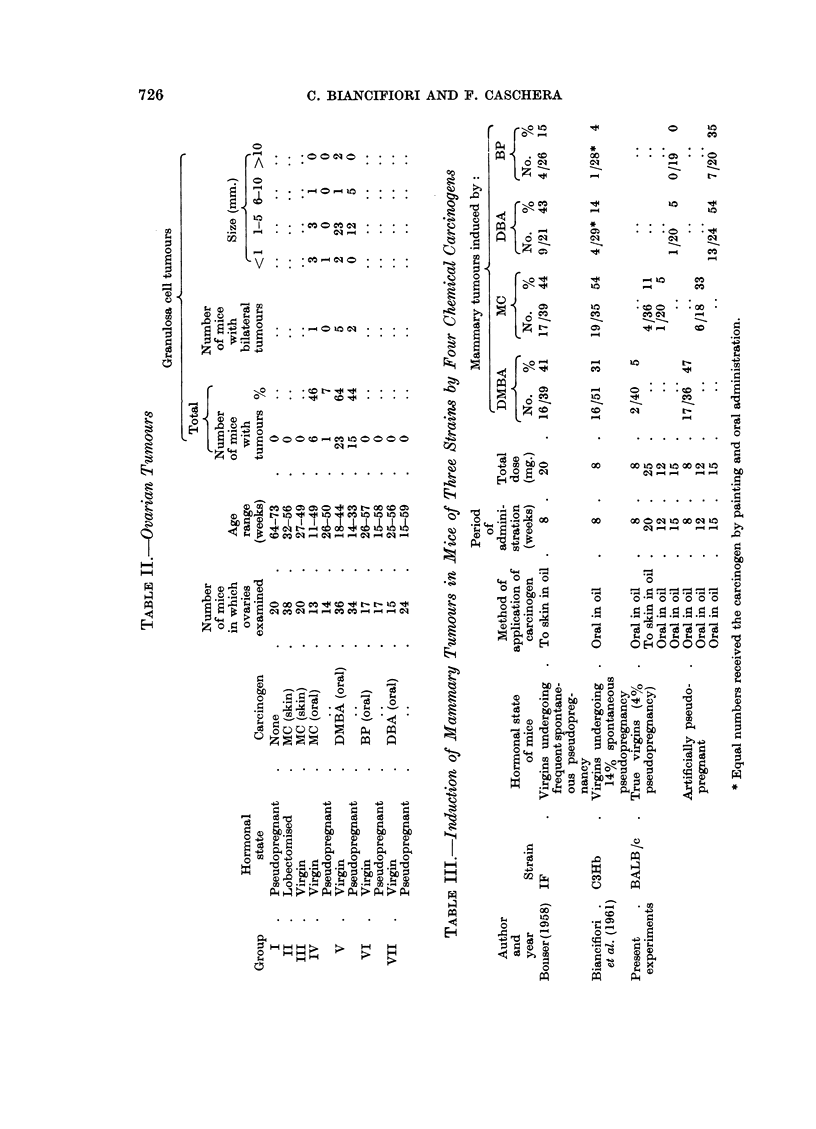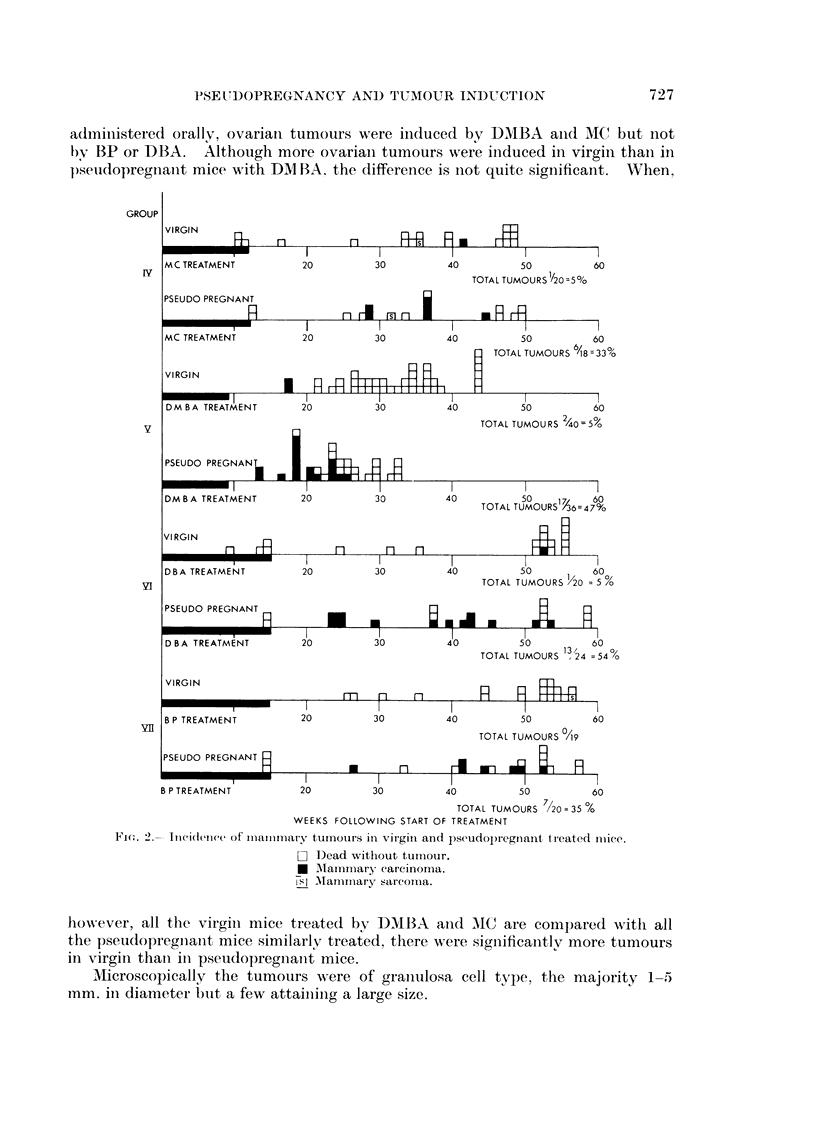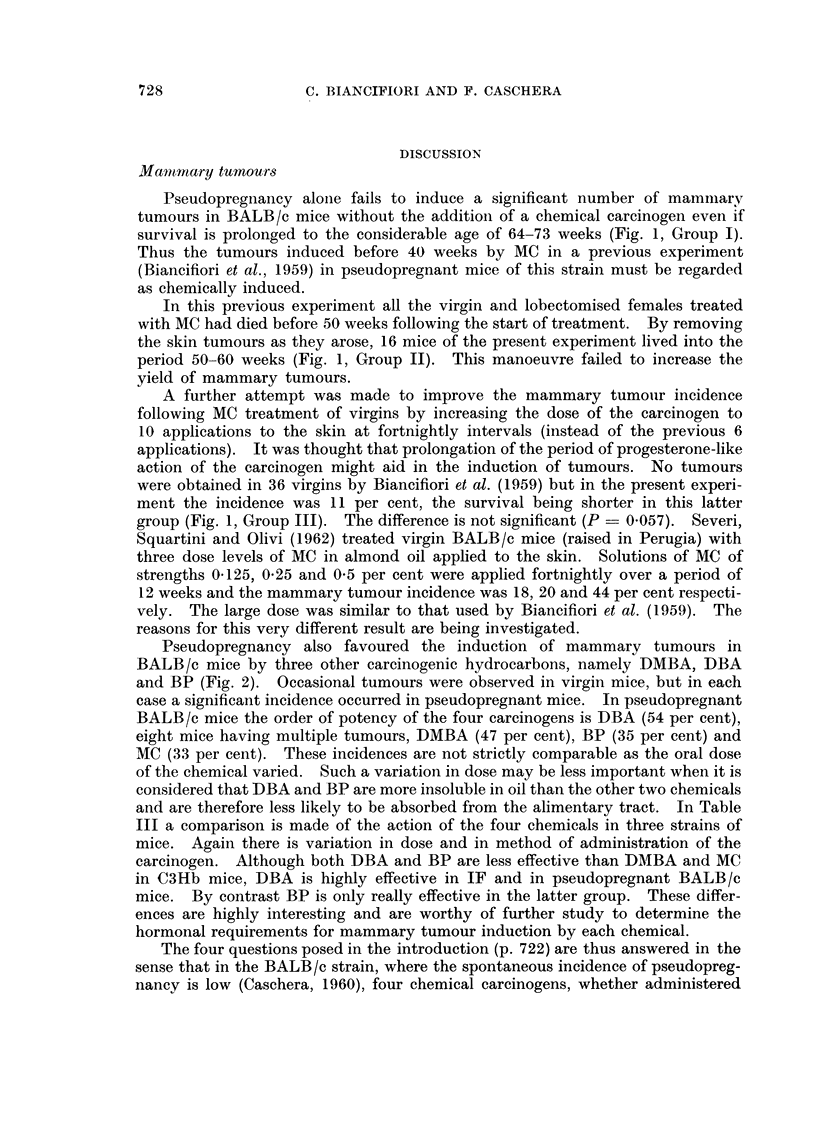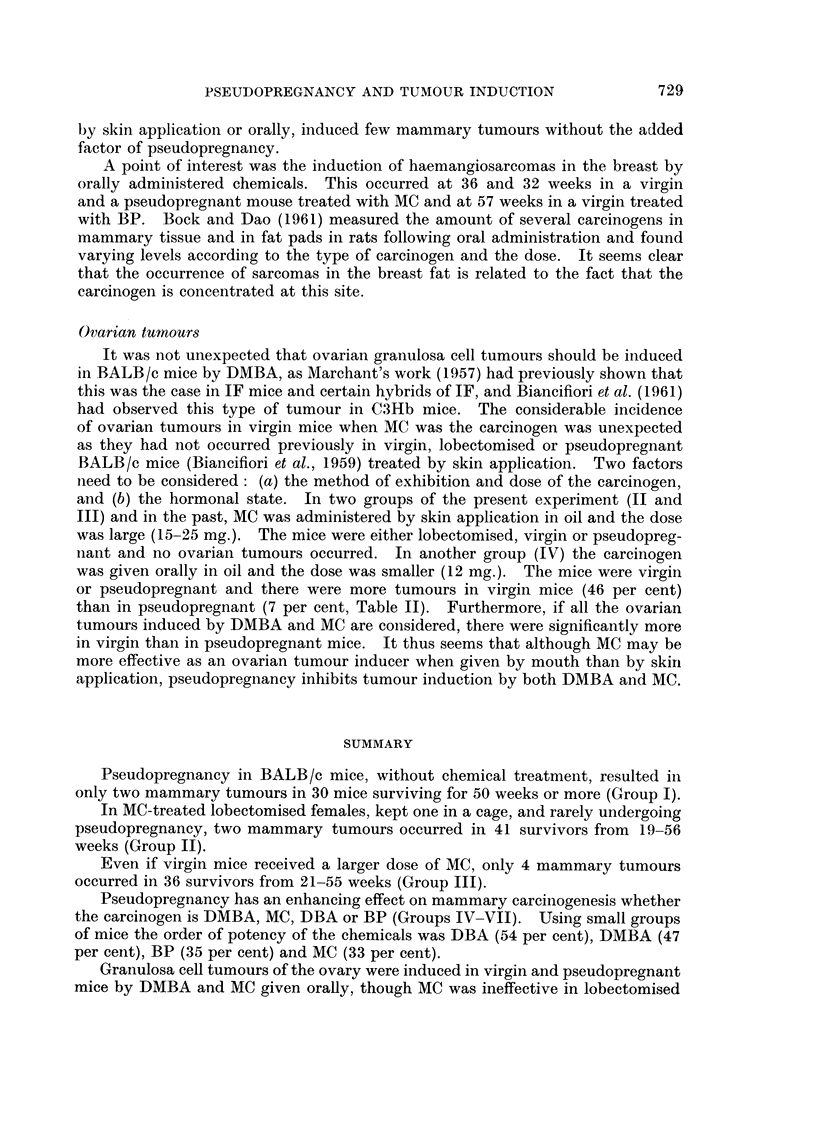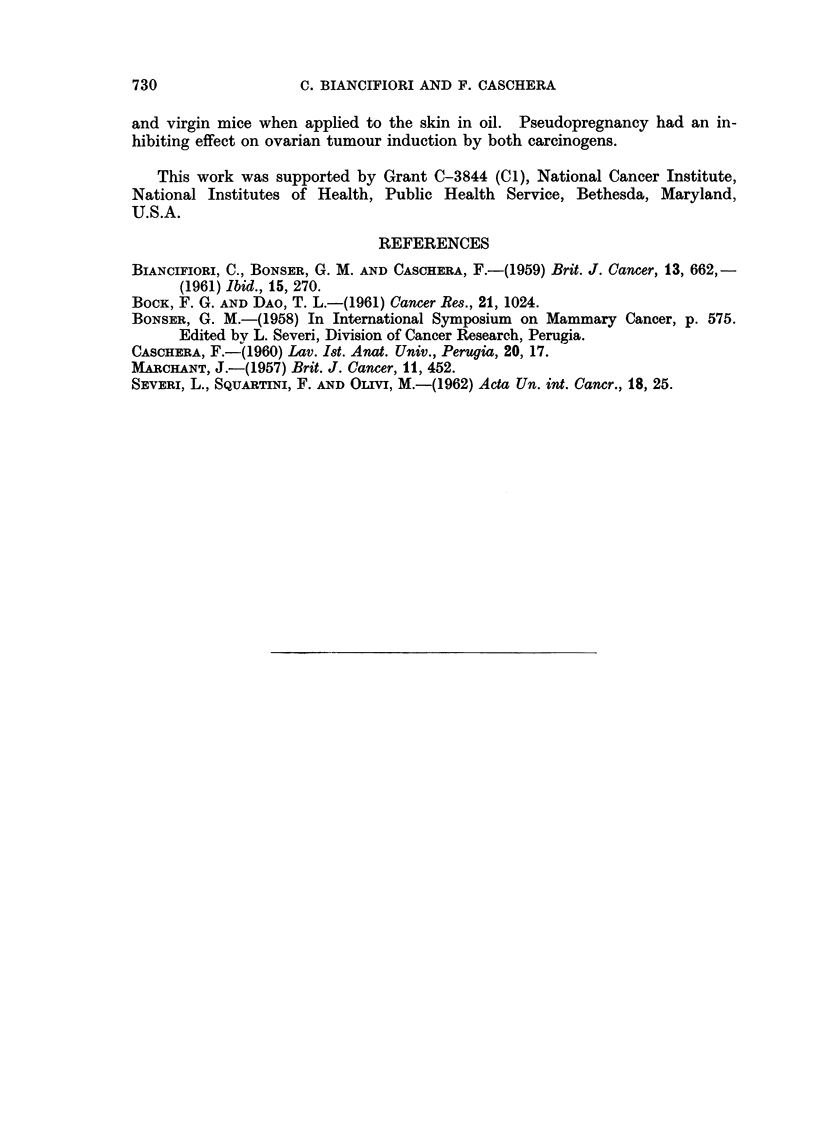# The Relation Between Pseudopregnancy and the Chemical Induction by Four Carcinogens of Mammary and Ovarian Tumours in BALB/c Mice

**DOI:** 10.1038/bjc.1962.84

**Published:** 1962-12

**Authors:** C. Biancifiori, F. Caschera


### THE RELATION BETWEEN PSEUDOPREGNANCY AND THE CHEMICAL INDUCTION BY FOUR CARCINOGENS OF MAMMARY AND OVARIAN TUMOURS IN BALB/C MICE

C. BIANCIFIORI AND F. CASCHERA

*From the Division of Cancer Research, University of Study, Perugia, Italy*

Received for publication October 4, 1962

Biancifiori, Bonser and Caschera (1959) showed that mammary carcinomas did not occur in BALB/c virgin or lobectomised female mice following skin application of 20-methylcholanthrene (MC) for a limited period of 12 weeks. Virgin BALB/c mice, even when caged 5 in a box, rarely undergo pseudopregnancy (Caschera, 1960) and do so less frequently when the olfactory lobes have been removed. By rendering BALB/c mice artificially pseudopregnant the incidence of mammary tumours following MC treatment was 44 per cent. Marked differences were observed in the structure of the unaffected breasts, uteri and ovaries in non-pseudopregnant and pseudopregnant mice, and thus it was concluded that the hormonal conditions of pseudopregnancy were requisite for the development of mammary cancer following limited chemical treatment of the strain. Ovarian tumours did not occur in any of the groups.

Bonser (1958) observed that a limited dose by skin application of the four carcinogens 9,10-dimethyl-1,2-benzanthracene (DMBA), MC, 1,2 : 5,6-dibenzanthracene (DBA) and 3,4-benzopyrene (BP) induced mammary tumours in virgin IF mice ; Biancifiori, Bonser and Caschera (1961) obtained a similar result in virgin C3Hb mice following limited oral administration. Both these strains undergo spontaneous pseudopregnancy ; and this was regarded as supplying the necessary hormonal state for mammary tumour induction.

The present experiments were designed to answer the following questions : (*a*) can pseudopregnancy alone, without chemical treatment, induce mammary cancer in BALB/c female mice ; (*b*) would mammary tumours occur in MC-treated lobectomised mice if survival could be prolonged beyond the 50 weeks of the previous experiments ; (*c*) would a larger dose of MC cause tumours in virgins and (*d*) is pseudopregnancy necessary for mammary tumour induction by the carcinogens DMBA, BP and DBA?

### MATERIAL AND METHODS

*BALB/c strain.*—The origin of these mice was described by Biancifiori *et al.* (1959). The mice used were in the 108th generation of inbreeding.

*Virgins.*—At 4–5 weeks of age, virgin females were placed 5 in a cage and were so kept throughout the experiment.

*Lobectomised.*—At approximately 6 weeks of age, under ether anaesthesia, the olfactory lobes were removed surgically. From the time of weaning onwards the mice were kept singly.

*Pseudopregnant.*—At 6 weeks of age, groups of three virgins were mated with one vasectomised male, which was allowed to remain in the cage throughout the experiment.

*Source of the carcinogens.*—All were bought from Messrs. Light & Co., Ltd., Colnbrook, Bucks.

*Skin application.*—0·5 per cent of the carcinogen was suspended in almond oil. The chosen number of applications were made to the skin at fortnightly intervals, 8 drops on the dorsal and 8 drops on the ventral surface, commencing at 12 weeks of age. A diet of cubes (supplied by NAFAG, Switzerland) and water *ad libitum* was given.

*Oral administration.*—0·5 per cent of the carcinogen was suspended in almond oil. The chosen dose was given once or twice weekly by stomach tube for varying periods.

### RESULTS

The experiments were performed as shown in Table I.

### *Mammary tumours*

*Group I, pseudopregnant but no chemical treatment.*—All the mice but three survived for 64–73 weeks (Fig. 1) and only two mammary carcinomas occurred (6·7 per cent) at 66 weeks of age. One had a regular tubular structure and one was composed of polygonal cells in solid columns.

*Group II, lobectomised, treated with skin application of MC for* 12 *weeks and skin tumours excised.*—Forty-one mice lived for 19 weeks or longer, 16 living for 51–56 weeks after the start of treatment (Fig. 1). Two mammary carcinomas occurred at 34 and 35 weeks (5 per cent) ; one was of irregular tubular and one of solid polygonal cell type, both with squamous metaplasia.

*Group III, virgin, treated with skin application of MC for* 20 *weeks.*—All the mice survived the treatment, but 19 died between 21 and 31 weeks and the remainder between 42 and 55 weeks (Fig. 1). Four mice bore mammary tumours at a late date (11·1 per cent). The tumours were of tubular or solid structure, two containing areas of squamous metaplasia. In addition, in three mice 28 weeks following the start of treatment there were small thickenings in the breast, noticed with the naked eye, which proved on microscopical examination to be nodules composed of a duct, with or without squamous metaplasia, surrounded by acini filled with secretion. The epithelium of the ducts and acini was more folded than normal, though of low cuboidal type and with pyknotic nuclei. The explanation of these nodules seems to be that proliferation had commenced and then had partially regressed, possibly due to the withdrawal of the progesterone-mimetic stimulation of the carcinogen at 20 weeks, i.e. 8 weeks previously.

When it had been decided to test other carcinogens as mammary tumour inducers, skin applications of DMBA were instituted. The mortality was so high that recourse was had to oral administration of smaller doses and this method was adopted for all four chemicals of the following groups.

*Groups IV–VII, virgin and pseudopregnant, treated with four oral carcinogens (DBA, DMBA, BP and MC).*—The difference in mammary carcinoma incidence in virgin and pseudopregnant mice was marked no matter which chemical was used (Fig. 2). If all the 197 mice are grouped together, 4 per cent of tumours

TABLE I.—*Description of Hormonal State and Carcinogen Treatment in Seven Groups of Female BALB/c Mice*

| Group | Number of mice and hormonal state | | | Carcinogen | Method of treatment | | Fortnightly dose (mg.) | Period of administration (weeks) | Total dose (mg.) |
|---|---|---|---|---|---|---|---|---|---|
| | Virgin | Lobectomised | Pseudopregnant | | Skin application | Stomach tube | | | |
| I | .. | .. | 30 | None | .. | .. | .. | .. | .. |
| II | .. | 41 | .. | MC | +* | .. | 2·5 | 12 | 15 |
| III | 36 | .. | .. | MC | + | .. | 2·5 | 20 | 25 |
| IV | 20 | .. | 18 | MC | .. | + | 2† | 12 | 12 |
| V | 20 | .. | 20 | DMBA | .. | + | 2 | 8 | 8 |
| | 20 | .. | 16 | DMBA | .. | + | 2† | 10 | 10 |
| IV | 20 | .. | 24 | DBA | .. | + | 2† | 15 | 15 |
| VII | 19 | .. | 20 | BP | .. | + | 2† | 15 | 15 |

* Cutaneous tumours removed surgically as required.

† 0·5 mg. twice per week.

occurred in virgins and 44 per cent in pseudopregnant mice. When the chemicals are considered separately, the order of potency in pseudopregnant mice is DBA (54 per cent), DMBA (47 per cent), BP (35 per cent) and MC (33 per cent). In addition to the higher incidence with DBA this was the only group in which there were multiple tumours (in 8 out of 13 tumour-bearing mice). On the other hand the latent period of tumour induction was earlier with DMBA than with DBA (Fig. 2).

No significant differences in tumour morphology were observed. All the carcinomas had the variable tubular or anaplastic structure of chemically-induced

FIG. 1.—Incidence of mammary tumours in untreated and MC-treated mice.
□ Dead without tumour. ■ Mammary carcinoma.

mammary tumours and squamous metaplasia was present in all the pseudopregnant groups. It was most marked when DMBA was the carcinogen (15 out of 17 tumours) and least marked with DBA (6 out of 16 tumours).

A haemangiosarcoma occurred in the fat pad of one virgin and one pseudopregnant mouse treated with MC and one virgin mouse treated with BP (Fig. 2).

*Ovarian tumours*

Although the primary interest of these experiments was the study of the mammary tumours, observation was also made of the occurrence of ovarian tumours (Table II). None were observed in untreated pseudopregnant mice (Group I), twenty of which survived from 40–73 weeks. Nor were there any ovarian tumours in Groups II and III, where MC was administered by skin application to lobectomised and virgin mice and where survival was long enough to allow for tumours to develop. In the remaining groups, where four carcinogens were

TABLE II.—*Ovarian Tumours*

| Group | Hormonal state | Carcinogen | Number of mice in which ovaries examined | Age range (weeks) | Granulosa cell tumours: Total: Number of mice with tumours | % | Number of mice with bilateral tumours | Size (mm.) <1 | 1–5 | 6–10 | >10 |
|---|---|---|---|---|---|---|---|---|---|---|---|
| I | Pseudopregnant | None | 20 | 64–73 | 0 | .. | .. | .. | .. | .. | .. |
| II | Lobectomised | MC (skin) | 38 | 32–56 | 0 | .. | .. | .. | .. | .. | .. |
| III | Virgin | MC (skin) | 20 | 27–49 | 0 | .. | .. | .. | .. | .. | .. |
| IV | Virgin | MC (oral) | 13 | 11–49 | 6 | 46 | 1 | 3 | 3 | 1 | 0 |
| | Pseudopregnant | .. | 14 | 26–50 | 1 | 7 | 0 | 1 | 0 | 0 | 0 |
| V | Virgin | DMBA (oral) | 36 | 18–44 | 23 | 64 | 5 | 2 | 23 | 1 | 2 |
| | Pseudopregnant | .. | 34 | 14–33 | 15 | 44 | 2 | 0 | 12 | 5 | 0 |
| VI | Virgin | BP (oral) | 17 | 26–57 | 0 | .. | .. | .. | .. | .. | .. |
| | Pseudopregnant | .. | 17 | 15–58 | 0 | .. | .. | .. | .. | .. | .. |
| VII | Virgin | DBA (oral) | 15 | 25–56 | 0 | .. | .. | .. | .. | .. | .. |
| | Pseudopregnant | .. | 24 | 15–59 | 0 | .. | .. | .. | .. | .. | .. |

TABLE III.—*Induction of Mammary Tumours in Mice of Three Strains by Four Chemical Carcinogens*

| Author and year | Strain | Hormonal state of mice | Method of application of carcinogen | Period of administration (weeks) | Total dose (mg.) | Mammary tumours induced by: DMBA No. | DMBA % | MC No. | MC % | DBA No. | DBA % | BP No. | BP % |
|---|---|---|---|---|---|---|---|---|---|---|---|---|---|
| Bouser (1958) | IF | Virgins undergoing frequent spontaneous pseudopregnancy | To skin in oil | 8 | 20 | 16/39 | 41 | 17/39 | 44 | 9/21 | 43 | 4/26 | 15 |
| Biancifiori *et al.* (1961) | C3Hb | Virgins undergoing 14% spontaneous pseudopregnancy | Oral in oil | 8 | 8 | 16/51 | 31 | 19/35 | 54 | 4/29* | 14 | 1/28* | 4 |
| Present experiments | BALB/c | True virgins (4% pseudopregnancy) | Oral in oil | 8 | 8 | 2/40 | 5 | .. | | .. | | .. | |
| | | | To skin in oil | 20 | 25 | .. | | 4/36 | 11 | .. | | .. | |
| | | | Oral in oil | 12 | 12 | .. | | 1/20 | 5 | .. | | .. | |
| | | | Oral in oil | 15 | 15 | .. | | .. | | 1/20 | 5 | 0/19 | 0 |
| | | Artificially pseudopregnant | Oral in oil | 8 | 8 | 17/36 | 47 | .. | | .. | | .. | |
| | | | Oral in oil | 12 | 12 | .. | | 6/18 | 33 | .. | | .. | |
| | | | Oral in oil | 15 | 15 | .. | | .. | | 13/24 | 54 | 7/20 | 35 |

* Equal numbers received the carcinogen by painting and oral administration.

administered orally, ovarian tumours were induced by DMBA and MC but not by BP or DBA. Although more ovarian tumours were induced in virgin than in pseudopregnant mice with DMBA, the difference is not quite significant. When,

FIG. 2.—Incidence of mammary tumours in virgin and pseudopregnant treated mice.

□ Dead without tumour.

■ Mammary carcinoma.

[S] Mammary sarcoma.

however, all the virgin mice treated by DMBA and MC are compared with all the pseudopregnant mice similarly treated, there were significantly more tumours in virgin than in pseudopregnant mice.

Microscopically the tumours were of granulosa cell type, the majority 1–5 mm. in diameter but a few attaining a large size.

### DISCUSSION

### *Mammary tumours*

Pseudopregnancy alone fails to induce a significant number of mammary tumours in BALB/c mice without the addition of a chemical carcinogen even if survival is prolonged to the considerable age of 64–73 weeks (Fig. 1, Group I). Thus the tumours induced before 40 weeks by MC in a previous experiment (Biancifiori *et al.*, 1959) in pseudopregnant mice of this strain must be regarded as chemically induced.

In this previous experiment all the virgin and lobectomised females treated with MC had died before 50 weeks following the start of treatment. By removing the skin tumours as they arose, 16 mice of the present experiment lived into the period 50–60 weeks (Fig. 1, Group II). This manoeuvre failed to increase the yield of mammary tumours.

A further attempt was made to improve the mammary tumour incidence following MC treatment of virgins by increasing the dose of the carcinogen to 10 applications to the skin at fortnightly intervals (instead of the previous 6 applications). It was thought that prolongation of the period of progesterone-like action of the carcinogen might aid in the induction of tumours. No tumours were obtained in 36 virgins by Biancifiori *et al.* (1959) but in the present experiment the incidence was 11 per cent, the survival being shorter in this latter group (Fig. 1, Group III). The difference is not significant ($P = 0{\cdot}057$). Severi, Squartini and Olivi (1962) treated virgin BALB/c mice (raised in Perugia) with three dose levels of MC in almond oil applied to the skin. Solutions of MC of strengths 0·125, 0·25 and 0·5 per cent were applied fortnightly over a period of 12 weeks and the mammary tumour incidence was 18, 20 and 44 per cent respectively. The large dose was similar to that used by Biancifiori *et al.* (1959). The reasons for this very different result are being investigated.

Pseudopregnancy also favoured the induction of mammary tumours in BALB/c mice by three other carcinogenic hydrocarbons, namely DMBA, DBA and BP (Fig. 2). Occasional tumours were observed in virgin mice, but in each case a significant incidence occurred in pseudopregnant mice. In pseudopregnant BALB/c mice the order of potency of the four carcinogens is DBA (54 per cent), eight mice having multiple tumours, DMBA (47 per cent), BP (35 per cent) and MC (33 per cent). These incidences are not strictly comparable as the oral dose of the chemical varied. Such a variation in dose may be less important when it is considered that DBA and BP are more insoluble in oil than the other two chemicals and are therefore less likely to be absorbed from the alimentary tract. In Table III a comparison is made of the action of the four chemicals in three strains of mice. Again there is variation in dose and in method of administration of the carcinogen. Although both DBA and BP are less effective than DMBA and MC in C3Hb mice, DBA is highly effective in IF and in pseudopregnant BALB/c mice. By contrast BP is only really effective in the latter group. These differences are highly interesting and are worthy of further study to determine the hormonal requirements for mammary tumour induction by each chemical.

The four questions posed in the introduction (p. 722) are thus answered in the sense that in the BALB/c strain, where the spontaneous incidence of pseudopregnancy is low (Caschera, 1960), four chemical carcinogens, whether administered by skin application or orally, induced few mammary tumours without the added factor of pseudopregnancy.

A point of interest was the induction of haemangiosarcomas in the breast by orally administered chemicals. This occurred at 36 and 32 weeks in a virgin and a pseudopregnant mouse treated with MC and at 57 weeks in a virgin treated with BP. Bock and Dao (1961) measured the amount of several carcinogens in mammary tissue and in fat pads in rats following oral administration and found varying levels according to the type of carcinogen and the dose. It seems clear that the occurrence of sarcomas in the breast fat is related to the fact that the carcinogen is concentrated at this site.

*Ovarian tumours*

It was not unexpected that ovarian granulosa cell tumours should be induced in BALB/c mice by DMBA, as Marchant's work (1957) had previously shown that this was the case in IF mice and certain hybrids of IF, and Biancifiori *et al.* (1961) had observed this type of tumour in C3Hb mice. The considerable incidence of ovarian tumours in virgin mice when MC was the carcinogen was unexpected as they had not occurred previously in virgin, lobectomised or pseudopregnant BALB/c mice (Biancifiori *et al.*, 1959) treated by skin application. Two factors need to be considered : (*a*) the method of exhibition and dose of the carcinogen, and (*b*) the hormonal state. In two groups of the present experiment (II and III) and in the past, MC was administered by skin application in oil and the dose was large (15–25 mg.). The mice were either lobectomised, virgin or pseudopregnant and no ovarian tumours occurred. In another group (IV) the carcinogen was given orally in oil and the dose was smaller (12 mg.). The mice were virgin or pseudopregnant and there were more tumours in virgin mice (46 per cent) than in pseudopregnant (7 per cent, Table II). Furthermore, if all the ovarian tumours induced by DMBA and MC are considered, there were significantly more in virgin than in pseudopregnant mice. It thus seems that although MC may be more effective as an ovarian tumour inducer when given by mouth than by skin application, pseudopregnancy inhibits tumour induction by both DMBA and MC.

### SUMMARY

Pseudopregnancy in BALB/c mice, without chemical treatment, resulted in only two mammary tumours in 30 mice surviving for 50 weeks or more (Group I).

In MC-treated lobectomised females, kept one in a cage, and rarely undergoing pseudopregnancy, two mammary tumours occurred in 41 survivors from 19–56 weeks (Group II).

Even if virgin mice received a larger dose of MC, only 4 mammary tumours occurred in 36 survivors from 21–55 weeks (Group III).

Pseudopregnancy has an enhancing effect on mammary carcinogenesis whether the carcinogen is DMBA, MC, DBA or BP (Groups IV–VII). Using small groups of mice the order of potency of the chemicals was DBA (54 per cent), DMBA (47 per cent), BP (35 per cent) and MC (33 per cent).

Granulosa cell tumours of the ovary were induced in virgin and pseudopregnant mice by DMBA and MC given orally, though MC was ineffective in lobectomised and virgin mice when applied to the skin in oil. Pseudopregnancy had an inhibiting effect on ovarian tumour induction by both carcinogens.

This work was supported by Grant C–3844 (C1), National Cancer Institute, National Institutes of Health, Public Health Service, Bethesda, Maryland, U.S.A.

### REFERENCES

Biancifiori, C., Bonser, G. M. and Caschera, F.—(1959) *Brit. J. Cancer*, **13**, 662,—(1961) *Ibid.*, **15**, 270.

Bock, F. G. and Dao, T. L.—(1961) *Cancer Res.*, **21**, 1024.

Bonser, G. M.—(1958) In International Symposium on Mammary Cancer, p. 575. Edited by L. Severi, Division of Cancer Research, Perugia.

Caschera, F.—(1960) *Lav. Ist. Anat. Univ., Perugia*, **20**, 17.

Marchant, J.—(1957) *Brit. J. Cancer*, **11**, 452.

Severi, L., Squartini, F. and Olivi, M.—(1962) *Acta Un. int. Cancr.*, **18**, 25.